# Putative risk alleles for LATE‐NC with hippocampal sclerosis in population‐representative autopsy cohorts

**DOI:** 10.1111/bpa.12773

**Published:** 2019-08-27

**Authors:** Suvi R. K. Hokkanen, Mia Kero, Karri Kaivola, Sally Hunter, Hannah A. D. Keage, Anna Kiviharju, Anna Raunio, Pentti J. Tienari, Anders Paetau, Fiona E. Matthews, Jane Fleming, Caroline Graff, Tuomo M. Polvikoski, Liisa Myllykangas, Carol Brayne, EClipSE Collaboration

**Affiliations:** ^1^ Institute of Public Health University of Cambridge Cambridge UK; ^2^ Department of Pathology University of Helsinki and HUSLAB, Helsinki University Hospital Helsinki Finland; ^3^ Molecular Neurology, Research Programs Unit University of Helsinki Helsinki Finland; ^4^ Department of Neurology Helsinki University Hospital Helsinki Finland; ^5^ Cognitive Ageing and Impairment Neurosciences Laboratory, School of Psychology, Social Work and Social Policy University of South Australia Adelaide Australia; ^6^ Institute for Health and Society Newcastle University Newcastle upon Tyne UK; ^7^ Division of Neurogeriatrics, Department of Neurobiology, Care Sciences and Society Karolinska Institutet J10:20, Visionsgatan 4 Solna 171 64 Sweden; ^8^ Theme Aging, Genetics Unit Karolinska University Hospital‐Solna QA22 Stockholm Sweden; ^9^ Institute of Neuroscience Newcastle University Newcastle upon Tyne UK

**Keywords:** ABCC9, GRN, hippocampal sclerosis, LATE‐NC, population study, TDP‐43, TMEM106B

## Abstract

Limbic‐predominant age‐related TAR‐DNA‐binding protein‐43 (TDP‐43) encephalopathy with hippocampal sclerosis pathology (LATE‐NC + HS) is a neurodegenerative disorder characterized by severe hippocampal CA1 neuron loss and TDP‐43‐pathology, leading to cognitive dysfunction and dementia. Polymorphisms in *GRN*, *TMEM106B* and *ABCC9* are proposed as LATE‐NC + HS risk factors in brain bank collections. To replicate these results in independent population‐representative cohorts, hippocampal sections from brains donated to three such studies (Cambridge City over 75‐Cohort [CC75C], Cognitive Function and Ageing Study [CFAS], and Vantaa 85+ Study) were stained with hematoxylin–eosin (n = 744) and anti‐pTDP‐43 (n = 713), and evaluated for LATE‐NC + HS and TDP‐43 pathology. Single nucleotide polymorphism genotypes in *GRN* rs5848, *TMEM106B* rs1990622 and *ABCC9* rs704178 were determined. LATE‐NC + HS (n = 58) was significantly associated with the *GRN* rs5848 genotype (χ^2^(2) = 20.61, *P* < 0.001) and T‐allele (χ^2^(1) = 21.04, *P* < 0.001), and *TMEM106B* rs1990622 genotype (Fisher's exact test, *P* < 0.001) and A‐allele (χ^2^(1) = 25.75, *P* < 0.001). No differences in *ABCC9* rs704178 genotype or allele frequency were found between LATE‐NC + HS and non‐LATE‐NC + HS neuropathology cases. Dentate gyrus TDP‐43 pathology associated with *GRN* and *TMEM106B* variations, but the association with *TMEM106B* nullified when LATE‐NC + HS cases were excluded. Our results indicate that *GRN* and *TMEM106B* are associated with severe loss of CA1 neurons in the aging brain, while *ABCC9* was not confirmed as a genetic risk factor for LATE‐NC + HS. The association between *TMEM106B* and LATE‐NC + HS may be independent of dentate TDP‐43 pathology.

## Introduction

Hippocampal sclerosis in old age is a dementing disorder characterized by severe loss of pyramidal neurons and gliosis in the hippocampal CA1 area. In addition to the anatomically defined neuron loss, hippocampal sclerosis is frequently associated with aggregations of transactive response DNA binding protein 43kDa (TDP‐43) 15. Recently, neuropathological changes of limbic‐predominant age‐related TDP‐43 encephalopathy (LATE) were recognized as disease entity 13. Hippocampal sclerosis in old age with TDP‐43 pathology is thus a subgroup of LATE‐NC (LATE‐NC + HS).

In three European population based studies of older people with brain donation, two from the UK and one from Finland, 79‐100% LATE‐NC + HS cases were found to have TDP‐43 pathology in the hippocampal dentate gyrus 6, 10. TDP‐43 pathology is also linked to hippocampal neuron loss of lesser extent than LATE‐NC + HS, indicating that TDP‐43 pathology without severe CA1 neuron loss may represent a precursor state for LATE‐NC + HS 6. The exact etiological pathophysiology of LATE‐NC + HS is still unclear, but polymorphisms in the progranulin‐encoding gene *GRN* (rs5848) 3, transmembrane protein 106B‐encoding gene *TMEM106B* (rs1990622) 16, and sulfonylurea receptor 2‐encoding *ABCC9* (rs704178, in near‐perfect linkage disequilibrium with rs704180) 14 have emerged as potential risk factors within US brain bank‐based genome‐wide association studies (GWAS).

*GRN* rs5848 TT genotype is associated with lower progranulin levels and has been linked with frontotemporal lobar degeneration with TDP‐43 positive inclusions (FTLD‐TDP) without *GRN* mutation 19. Progranulin has direct neurotrophic and inflammatory response‐modulating functions, and is found to play a role in TDP‐43 processing 22. *TMEM106B* is reported to modulate progranulin levels 4. *ABCC9* has not been associated with FTLD‐TDP, TDP‐43 accumulation or other neurodegenerative diseases than LATE‐NC + HS, and possible mechanisms of *ABCC9* variation affecting CA1 neuron loss are unclear 17.

No population‐representative analyses on *GRN* rs5848, *TMEM106B* rs1990622 and *ABCC9* rs704178 variants in LATE‐NC + HS have been published. Here, we investigate these proposed risk variations in three population‐representative cohorts of older people, and evaluate their association with LATE‐NC + HS, which could indicate the underlying pathophysiological mechanisms for CA1 neuron loss in the aging brain. We also assess if LATE‐NC without hippocampal sclerosis is associated with the proposed genetic variations.

## Methods

### Study design

**Figure 1:**
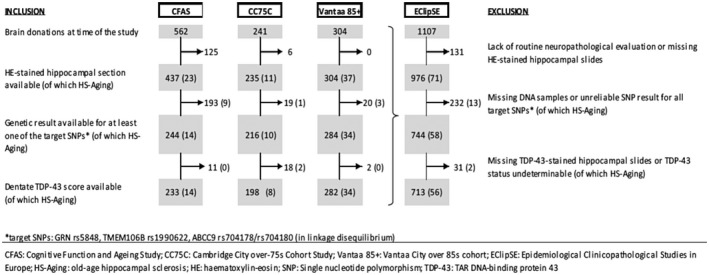
Attrition table indentifying the numbers of samples included in the study by cohort.

This study is set within EClipSE (Epidemiological Clinicopathological Studies in Europe), which is a collaboration of the three European population‐representative longitudinal cohort studies with brain donation programs: the Cognitive Function and Ageing Studies (CFAS) (multicenter, UK), the Cambridge City over‐75s Cohort (CC75C, Cambridge, UK), and the Vantaa 85+ study (Vantaa, Finland). Cohort profiles, recruiting procedures and the approach for brain donation have been described previously in detail 2, 5, 18. The CC75C had 2160 participants aged ≥75 years at baseline in 1985, CFAS recruited 18226 people aged ≥65 years in 1989–1993, and the Vantaa 85+ study comprised 601 people aged ≥85 years in 1991. In CC75C and CFAS, a stratified random subsample of the cohort was approached for brain donation, resulting in 11% donation in CC75C (n = 241 at the time of the study) and 3% donation in CFAS (n = 562) with known representation of the source population. In the Vantaa 85+ study, a consented autopsy was performed on 51% (n = 304) of the cohort. Of these 1107 EClipSE donations, 131 were excluded due to lack of routine neuropathological evaluation (i.e. donations were too recent) or missing hematoxylin–eosin‐stained hippocampal slides with used‐up corresponding tissue blocks, and further 232 donations were excluded due to missing or unreliable deoxyribonucleic acid (DNA) samples (Figure 1). Thus, 744 EClipSE samples (CC75C n = 216, CFAS n = 244, Vantaa 85+ n=284) are included in the current study (Figure 1). All data collection, including brain donation, followed local ethical guidelines and approval.

### Neuropathological protocol

In CFAS and CC75C, brains were removed as soon as possible after death and bisected. One hemisphere was then dissected coronally and frozen at − 80°C. The contralateral hemisphere was formalin‐fixed for at least six weeks, cut in coronal plane, and approximately 1‐centimeter (cm)‐thick samples were paraffin‐embedded. In Vantaa 85+, both hemispheres were formalin‐fixed, and dissected coronally. The right hemisphere was sampled for paraffin‐embedding, while samples from the left hemisphere were conserved in polyethylene glycol (PEG). PEG‐conserved hippocampal tissue was reprocessed into paraffin in 2013.

### Hippocampal sclerosis

Six‐ to nine‐micrometer (µm)‐thick hippocampal sections were stained with hematoxylin–eosin. To standardize the diagnosis of hippocampal sclerosis across all three EClipSE cohorts, available sections were systematically assessed for severe neuron loss by an inter‐rater‐controlled method 6. Hippocampal sclerosis was diagnosed as present or absent based on severe neuron loss (<5 neurons/ field of view [fov] at 200x magnification) in over half of CA1 fov, gliosis, neuronal loss not being explained by an infarct, and no obvious neuron loss in other hippocampal areas. Hippocampal sclerosis was present in 10/216 CC75C and in CFAS 14/224 samples (Figure 1). In the Vantaa 85+ study, sections were scored hippocampal sclerosis positive when at least one hemisphere met the hippocampal sclerosis criteria (n = 37) (Figure 1). It is of note that due to the standardization method used in this study, the number of hippocampal sclerosis cases in the Vantaa 85+ cohort identified is lower than that in the previously published study which used different diagnostic criteria 10.

### TDP‐43 immunohistochemistry and evaluation

In CC75C and CFAS, 431/460 samples were available for TDP‐43 assessment, while previous research had exhausted the hippocampal area on the remainder. Nine micrometer hippocampal sections were immunostained with anti‐pTDP‐43 antibody (pSer409/410‐2, polyclonal, CosmoBio, Japan) 9 at the Cambridge Brain Bank UK.

In Vantaa 85+, 282/284 samples were included in the TDP‐43 assessment. Hippocampal tissue sections (4μm) from the right hemisphere were immunostained with anti‐pTDP‐43 antibodies (pSer409/410, clone 11‐9, CosmoBio, Japan) 10.

Anti‐pTDP‐43 immunoreactive solid neuronal cytoplasmic inclusions (NCI) were evaluated blind to clinical or other pathological information as present or absent in the hippocampal dentate cell layer. In all three cohorts, TDP‐43 assessment was inter‐rater evaluated.

**Figure 2:**
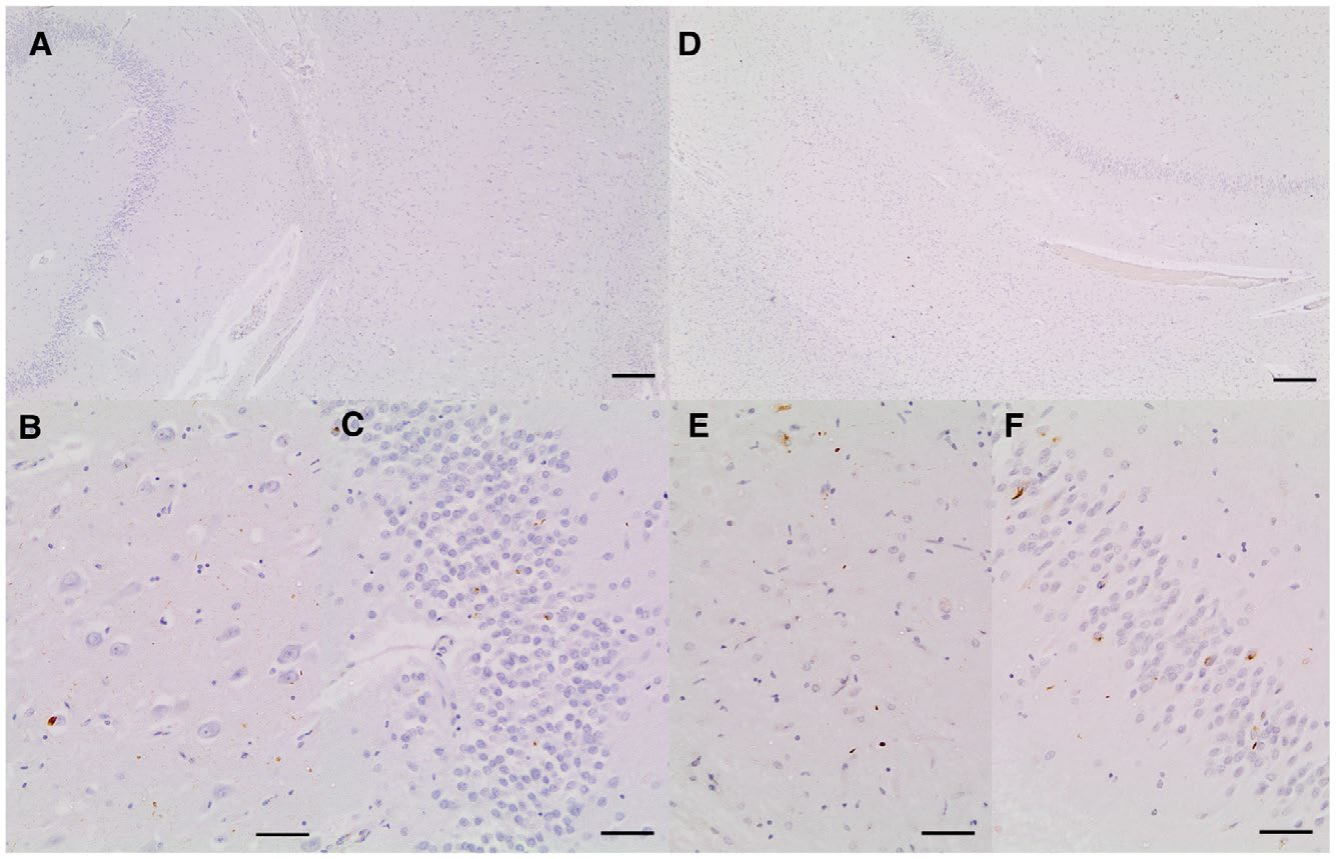
**A.** Section of hippocampus showing dentate, molecular layer and CA1 from a case with severe dentate neuronal inclusions but minimal cell loss in CA1. **B**. CA1 from same slide showing a range of pathologies immunoreactive for anti‐phosphorylated TDP‐43 antibody including cytoplasmic inclusions and neurites. **C.** Dentate from same slide showing a range of pathologies immunoreactive for antiphosphorylated TDP‐43 antibody including cytoplasmic inclusions and neurites. **D**. Section of hippocampus showing dentate, molecular layer and CA1 from a case with severe dentate neuronal inclusions and severe cell loss in CA1 qualifying as HScl. **E.** CA1 from same slide showing a range of pathologies immunoreactive for anti‐phosphorylated TDP‐43 antibody including cytoplasmic inclusions and neurites. **F**. Dentate from same slide showing a range of pathologies immunoreactive for anti‐phosphorylated TDP‐43 antibody including cytoplasmic inclusions and neurites. Scale bars: **A**,**D** = 200 μm, **B**,**C**,**E**,**F** = 50 μm.

LATE‐NC presents with TDP‐43 pathology in the amygdala at stage 1, and in the hippocampus at stage 2. Figure 2 illustrates typical hippocampal LATE‐NC TDP‐43 pathology without hippocampal sclerosis (Figure 2A‐C) and with hippocampal sclerosis (Figure 2D‐F). Dentate NCI pathology is highlighted in Figure 2C and F, respectively. In this study, dentate cell layer NCI TDP‐43 pathology was considered as LATE‐NC, because TDP‐43‐stained amygdala sections were not available across the EClipSE cohort. Moreover, the dentate cell layer has previously been staged as the first hippocampal region affected by TDP‐43 pathology after the subiculum 7. In cases with hippocampal sclerosis, CA1 and subiculum neuron loss may also be so complete that no NCI are visible in these regions. Figure S1 shows a LATE‐NC case with dentate TDP‐43 pathology but minimal CA1 pathology without hippocampal sclerosis (Figure S1 A‐C) and with hippocampal sclerosis (Figure S1D‐F).

### SNP analysis

Single nucleotide polymorphism (SNP) analysis for CC75C/CFAS was done at the Karolinska Institute, Sweden. A TaqMan SNP 7500 genotyping assay on real time PCR (Applied Biosystems, CA, USA) was used to determine the target SNPs. The target polymorphisms were *GRN* rs5848, *TMEM106B* rs1990622 and *ABCC9* rs704178. Pre‐designed TaqMan SNP Genotype Assays were available from ThermoFischer Scientific. For 19 CC75C subjects, DNA was extracted from paraffin‐embedded blocks, as no frozen tissue was available.

In the Vantaa 85+ cohort, genotypes of *GRN* rs5848 and *TMEM106B* rs1990622 were determined from whole‐genome sequencing (WGS) data in 150 subjects 11. Sanger sequencing was performed in the remainder samples. Variant containing sequences were first amplified using primers listed in Table 1. Fragments were run on an ABI3730xl DNA Analyzer at the Institute of Molecular Medicine Finland and the sequencing data were analyzed using Sequencher 4.0 analysis software (Applied Biosystems). Genotypes for *ABCC9* rs704180 were determined from the WGS data in 150 subjects and the remainder samples were Sanger sequenced for this SNP (data not shown). Genotype for ABCC9 rs704178 was imputed with Beagle 4.1 (version 27Jan18.7e1) using the population‐specific SISu v3 imputation reference panel (https://doi.org/10.17504/protocols.io.nmndc5e). The quality of imputed rs704178 genotypes was high and passed post‐imputation quality control (INFO score = 1, similar MAF in our imputed dataset and in the Finnish GnomAD population (0.44 vs. 0.41) and 98.6 % concordance between imputed and whole‐genome sequencing‐derived genotypes of internal control samples). The number of subjects with reliable results for each target SNP is reported in Table 2.

**Table 1:** PCR primers for Sanger sequencing. PCR = Polymerase chain reaction; SNP = single‐nucleotide polymorphism.

SNP	Gene	Forward primer (5′–3′)	Reverse primer (5′–3′)
rs5848	*GRN*	GCCAGGGGTACCAAGTGTTT	GCAGGGCGGCAAATCAGA
rs1990622	*TMEM106B*	ACACACGGCATTGTGTTTGATT	TGAGATGACCAGCCACTCCA

**Table 2:** Demographic characteristics of the cohort, and association of *GRN* rs5848, *TMEM106B* rs1990622 and *ABCC9* rs704178 with LATE‐NC + HS or dentate TDP‐43 solid neuronal inclusions. LATE‐NC + HS = Limbic‐predominant age‐related TDP‐43 encephalopathy neuropathological changes with hippocampal sclerosis; TDP‐43 = transactive response DNA binding protein 43 kDa; SD = standard deviation; n = number; MOI = mode of inheritance.

		No HS‐Aging (n = 686)	LATE‐NC + HS (n = 58)	Statistic	No dentate TDP‐43 NCI (n = 543)	Dentate TDP‐43 NCI (n = 170)	Statistic
Female (%)		69.1	91.4	χ^2^(1) = 12.85	70.2	75.9	χ^2^(1) = 2.08
				*P* < 0.001			*P* = 0.149
Age at death, mean ± SD (years)		89.7 ± 6.2	93.3 ± 4.8	t(742) = −4.33	89.5 ± 6.3	92.2 ± 4.9	t(711) = −5.20
				*P* < 0.001			*P* < 0.001
GRN rs5848							
Genotype, n (%)	C/C	342 (51.3)	12 (21.1)	χ^2^(2) = 20.61	280 (53.3)	58 (34.3)	χ^2^(2) = 18.59
	C/T	259 (38.8)	33 (57.9)	*P* < 0.001	193 (36.8)	86 (50.9)	*P* < 0.001
	T/T	66 (9.9)	12 (21.1)	phi = 0.169	52 (9.9)	25 (14.8)	phi = 0.164
Allelic frequency, (%)	C	70.7	50	χ^2^(1) = 21.04	71.7	59.8	χ^2^(1) = 17.01
				*P* < 0.001			*P* < 0.001
	T	29.3	50	phi = 0.121	28.3	40.2	phi = 0.111
TMEM106B rs1990622							
Genotype, n (%)	A/A	240 (36.6)	38 (66.7)	FET	186 (36.1)	81 (48.2)	χ^2^(2) = 10.33
							*P* = 0.006
	A/G	313 (47.7)	18 (31.6)	*P* < 0.001	245 (47.5)	72 (42.9)	
	G/G	103 (15.7)	1 (1.8)	phi = 0.176	85 (16.5)	15 (8.9)	phi = 0.123
Allelic frequency, (%)	A	58.2	82.5	χ^2^(1) = 25.75	59.8	69.6	χ^2^(1) = 10.47
				*P* < 0.001			*P* = 0.001
	G	41.8	17.4	phi = 0.123	40.2	30.4	phi = 0.088
ABCC9 rs704178							
Genotype, n (%)	C/C	137 (20.0)	7 (12.3)	χ^2^(2) = 3.20	114 (21.0)	27 (16.0)	χ^2^(2) = 2.57
	C/G	354 (51.7)	36 (63.2)	*P* = 0.202	282 (52.0)	89 (52.7)	*P* = 0.276
	G/G	194 (28.3)	14 (24.6)	phi = 0.066	146 (26.9)	53 (31.4)	phi = 0.060
*Recessive MOI*	C/C, C/G	491 (71.7)	43 (75.4)	χ^2^(2) = 0.37	396 (73.1)	116 (68.6)	χ^2^(2) = 1.25
				*P* = 0.544			*P* = 0.263
	G/G	194 (28.3)	14 (24.6)	phi = −0.022	146 (26.9)	53 (31.4)	phi = 0.042
Allelic frequency, (%)	C	45.8	43.9	χ^2^(1) = 0.16	47	42.3	χ^2^(1) = 2.33
				*P* = 0.683			*P* = 0.127
	G	54.2	56.1	phi = −0.011	53	57.7	phi = −0.041

For the EClipSE cohort, G/C SNP rs704178 of gene *ABCC9* were determined, because the genotype data were not available for recommended reference A/G SNP rs704180 of all three studies. However, these two SNPs are in near perfect linkage disequilibrium.

### Statistical approach

For CFAS and CC75C samples, TDP‐43 inter‐rater agreement was assessed calculating Gwet's Agreement Coefficient 2 (AC2) using Agreestat 2015.1 (Advanced Analytics, Gaithersburg, MD, USA). Near‐perfect agreement was reached for all inter‐rater evaluations (CC75C: AC2 = 0.99, 95% Confidence Interval [CI]: 0.97–1; CFAS: AC2 = 0.98, 95% CI: 0.96–1). For Vantaa 85+ samples, TDP‐43 pathologies were confirmed by a second and a third rater.

Hardy–Weinberg equilibrium (HWE) expected frequencies were calculated and tested using Chi2 goodness of fit (χ^2^(degree of freedom [df])). Chi2 (χ^2^(df)) (or Fisher's exact test [FET] when appropriate) was used to test the association of genotype or allele frequency and LATE‐NC + HS or TDP‐43 pathology. Each SNP was analyzed using an additive mode of inheritance (number of risk alleles, ie, 0, 1, or 2). *ABCC9* rs704178 was furthermore analyzed using a recessive mode of inheritance (2 risk alleles = 1; 0 otherwise), as this has previously been shown to be appropriate 8, 14, 16. Effect size for Cramér's phi is reported. Logistic regression (Odds ratio [OR], 95% CI) was used to test genotype associations with LATE‐NC + HS or TDP‐43 pathology when taking the effect of age at death and sex into consideration. α was set at 0.05. Data were analyzed using STATA14 software (Stata Corporation 2015, Texas, USA).

## Results

### Hippocampal sclerosis and TDP‐43

All hippocampal sclerosis cases in CC75C and CFAS with available TDP‐43 staining (n = 22) were positive for hippocampal dentate NCI, while in the Vantaa 85+ cohort 26 of the 34 hippocampal sclerosis cases with TDP‐43‐immunostained sections presented with dentate NCI. However, in the Vantaa 85+ cohort, hippocampal sclerosis was evaluated bilaterally, whereas TDP‐43 was evaluated only on the right hemisphere. All Vantaa 85+ cases with hippocampal sclerosis pathology on the right hemisphere were positive for dentate TDP‐43 NCI on the ipsilateral side. The eight Vantaa 85+ hippocampal sclerosis cases which were negative for dentate TDP‐43 NCI presented with unilateral hippocampal sclerosis on the left hemisphere. Therefore, these cases cannot be considered as dentate TDP‐43 NCI negative, and all hippocampal sclerosis cases in the EClipSE cohort are referred to as LATE‐NC + HS in this study.

### LATE‐NC + HS, genes and TDP‐43

Table 2 presents demographic factors, *GRN, TMEM106B* and *ABCC9* genotype frequencies, as well as LATE‐NC + HS and dentate TDP‐43 NCI prevalence for the EClipSE cohort. The genotype frequencies did not deviate from HWE.

### *GRN* and LATE‐NC + HS

LATE‐NC + HS associated significantly with the *GRN* rs5848 genotype in EClipSE cohorts (χ^2^(2) = 20.61, *P* < 0.001, phi = 0.169), presenting more frequently with C/T and T/T genotypes compared to the remainder population (Table 2). This association remained when adjusting for sex and age at death in logistic regression (OR: 2.97, 95% CI: 1.77–4.98, *P* < 0.001). LATE‐NC + HS cases were significantly associated with the risk‐allele T in the EClipSE cohorts (χ^2^(1) = 21.04, *P* < 0.001, phi = 0.121).

### *TMEM106B* and LATE‐NC + HS

LATE‐NC + HS was significantly associated with the *TMEM106B* genotype in EClipSE (FET *P* < 0.001, phi = 0.176, Table 2), also when adjusting for sex and age at death in logistic regression (OR: 0.31, 95% CI: 0.17–0.54, *P* < 0.001). LATE‐NC + HS cases presented more frequently with the genotype A/A than the remainder population (χ^2^(1) = 25.75, *P* < 0.001, phi = 0.123, Table 2).

### *ABCC9* and LATE‐NC + HS

LATE‐NC + HS did not differ from the remainder population in *ABCC9* rs704178 genotype or allele frequency, irrespective of the analyzed mode of inheritance (additive or recessive) (Table 2). Controlling for sex and age at death in logistic regression did not influence the result (additive MOI: OR: 1.06, 95% CI: 0.59–1.95, *P* = 0.824; recessive MOI: OR: 0.84, 95% CI: 0.44–1.59, *P* = 0.593).

### LATE‐NC + HS risk genes and TDP‐43 pathology

Presence of dentate TDP‐43 NCI was significantly associated with the *GRN* rs5848 genotypes T/C and T/T (χ^2^(2) = 18.59, *P* < 0.001, phi = 0.164), also when controlling for sex and age at death in logistic regression (OR: 1.68, 95% CI: 1.30–2.17, *P* < 0.001), and the T‐allele (χ^2^(1) = 17.01, *P* < 0.001, phi = 0.111). Dentate TDP‐43 NCI was also significantly associated with the T*MEM106B* rs1990622 genotype A/A (χ^2^(2) = 10.33, *P* = 0.006, phi = 0.123), even when accounted for sex and age at death (OR: 0.67, 95% CI: 0.51–0.88, *P* = 0.004), and the A‐allele (χ^2^(1) = 10.47, *P* = 0.001, phi = 0.088) (Table 2). The distribution of *ABCC9* rs704178 genotype and allele did not differ between EClipSE subjects with dentate TDP‐43 NCI pathology compared to those without (Table 2). The association remained insignificant when controlling for sex and age at death in logistic regression (additive MOI: OR: 1.15, 95% CI: 0.73–1.81, *P* = 0.537; recessive MOI: OR: 1.29, 95% CI: 0.88–1.91, *P* = 0.187).

As all LATE‐NC + HS cases were of TDP‐43 positive or unknown status ipsilateral to the hippocampal sclerosis pathology, analyses on the association of dentate TDP‐43 NCI and LATE‐NC + HS risk genes were not conducted stratified by LATE‐NC + HS.

The association between dentate TDP‐43 NCI and the proposed LATE‐NC + HS risk genes was however evaluated while excluding LATE‐NC + HS cases. Dentate TDP‐43 NCI remained significantly associated with *GRN* rs5848 genotypes T/C and T/T (χ^2^(2) = 7.99, *P* = 0.018, phi = 0.112) and the *GRN* rs5848 T‐allele (χ^2^(1) = 7.6, *P* = 0.006, phi = 0.077) (Table S1), but the association between T*MEM106B* rs1990622 genotype or allele and dentate TDP‐43 NCI became insignificant when LATE‐NC + HS cases were not included in the analyses (Table S1).

## Discussion

Previous research, based on US brain bank collections drawing on volunteer and clinical dementia cohorts, has indicated that polymorphisms in *GRN*, *TMEM106B* and *ABCC9* genes are associated with hippocampal sclerosis in old age/LATE‐NC + HS, but to date no population‐representative data have been published. Here, we studied three European population‐representative cohorts and confirmed an association of LATE‐NC + HS with polymorphisms in *GRN* and *TMEM106B*. The published association of LATE‐NC + HS with *ABCC9* polymorphism could not be reproduced. These detected associations of *GRN* and *TMEM106B* with LATE‐NC + HS may be related to progranulin expression, as literature suggests both *GRN* and *TMEM106B* polymorphisms to be associated with variation in progranulin levels.

Limitations of this study include the overall cohort's sample size, different TDP‐43 antibodies and section thickness between the UK and Finnish cohorts, unilateral instead of bilateral assessment of hippocampal sclerosis in the UK studies and of TDP‐43 in all three cohorts, as well as lack of proof of causality. It is likely that we did not capture all LATE‐NC cases in these cohorts as TDP‐43 presence was only evaluated unilaterally in the hippocampal dentate. Further, not all hippocampal sclerosis cases could be verified as LATE‐NC as TDP‐43 staining was not available for each. Even though the EClipSE collaboration is the largest population‐representative epidemiological cohort with brain donation, numbers of LATE‐NC + HS cases may still be low for a genetic study. The lack of confirmation of the *ABCC9* locus must therefore be interpreted with caution. However, as our population‐representative study results give very strong independent support to the previously reported associations between LATE‐NC + HS and the polymorphic sites in *GRN* and *TMEM106B*, it seems likely that functions of these two genes indeed do affect survival of the CA1 neurons, at least in older individuals. On the other hand, to confirm the hypothesis that variations in *GRN* and *TMEM106B* contribute to LATE‐NC + HS neuron loss by altered progranulin levels, further studies would need to assess the expression of progranulin in association with LATE‐NC + HS.

Important strengths of this study are the high mean age and population‐representative origin of the cohorts, introducing minimal selection bias. Studies that previously reported polymorphisms in *GRN*, *TMEM106B* and *ABCC9* as risk factors for LATE‐NC + HS, obtained cases from brain banks and Alzheimer's Disease Centers 1, 8, 14, 16, which tend to oversample people with dementia and frequently lack sufficient numbers of the oldest old to test hypotheses in this age group. Results from these studies may therefore not be transferable to the general population. Moreover, one of the pitfalls in GWAS studies is multiple testing: many candidate polymorphisms are assessed, so associations may be significant by chance, even though their clinical meaning is not clear. Independent replication of positive associations is crucial. Results from GWAS which identified proposed LATE‐NC + HS risk genes 1, 14 have been replicated up to now only using similar sources 16. This study's results are the first to verify or challenge previous results in an independent cohort, including samples from three population‐representative cohorts from two countries.

Progranulin (encoded by gene *GRN* at chromosome 17) is universally expressed in the central nervous system (CNS) during early neuronal development, and is also expressed at modest levels in neurons and microglia in adults 21. Progranulin is proteolytically broken down into granulin peptides by extracellular proteases, mainly elastase, produced likely by astrocytes and microglia 3. Progranulin expression is markedly increased by neuroinflammation in association with neurodegenerative disease, where it appears to play a modulatory role in tissue damage within the CNS to suppress excessive immunity‐based microglial activation and protecting neurons from reactive oxygen species and proinflammatory cytokines 21. Most currently known *GRN* mutations causing FTLD‐TDP have been reported to create null alleles via haploinsufficiency mechanism, resulting in reduced protein levels 17. Progranulin may act as a potential neurotrophic factor whose loss of function may cause neurodegeneration in FTLD‐TDP or other related diseases, and lead to the development of TDP‐43‐positive pathology in LATE‐NC + HS 3. Progranulin expression may have effects on cleavage and distribution of TDP‐43 8. Notably, the polymorphic rs5848 site in the 3'‐untranslated region of *GRN*, which is associated with variations in progranulin levels and increased risk of LATE‐NC + HS, is also part of a binding site for microRNA miR‐659 19. miR‐659 may confer an increased risk for FTLD‐TDP and LATE‐NC + HS through an inhibition of progranulin translation, generating an effect resembling the biochemical and pathological findings observed in *GRN*‐null mutations 19, 21.

Gene *TMEM106B* (at chromosome 7p21) encodes a transmembrane protein, with a preferential expression in the frontal lobe 4. Genetic variation in *TMEM106B* may specifically modify the development of FTLD in the presence of a *GRN* mutation 12. The *TMEM106B* rs1990622 variant, where the C allele regulates *GRN* expression 4, is reported to be protective for LATE‐NC + HS within a cohort of Alzheimer's disease cases 20. Results from this study confirmed *GRN* rs5848 and *TMEM106B* rs1990622 to be associated with LATE‐NC + HS. Reduced progranulin levels could increase the vulnerability of specific CA1 neuron populations, through higher susceptibility for stress and inflammatory changes. As all LATE‐NC + HS cases presented with ipsilateral dentate TDP‐43 NCI, it was not possible to analyze the association between LATE‐NC + HS and the risk genes independent of TDP‐43. However, the association between *TMEM106B* and dentate TDP‐43 NCI was nullified when LATE‐NC + HS cases were excluded from the analyses, which indicates that the association between LATE‐NC + HS and *TMEM106B* is dependent on LATE‐NC + HS.

Previous studies evaluating the effect of *GRN* rs5848, *TMEM106B* rs1990622 and progranulin levels/lysosomal pathways on neurodegeneration were largely based on younger people than those who are at greatest risk of LATE‐NC + HS 9. It is conceivable that progranulin alterations might be tolerated at younger age, but that with increased age in LATE‐NC + HS, deterioration of cellular defense and clearing mechanisms lower this threshold, leading to CA1 neuron damage.

*ABCC9* gene encodes an evolutionarily conserved large polypeptide sulfonylurea receptor 2 (SUR2) with multiple membrane‐spanning domains and multiple levels of biologic complexity 17. Intronic SNPs that cluster in the 3′ portion of *ABCC9* have been associated with a risk for human brain illnesses, including sleep disturbances and LATE‐NC + HS 16. SUR2 transcript variants might have novel alternative splicing in the mRNAs' coding region and 3′ untranslated region (3′UTR) variants 17. The claimed LATE‐NC + HS risk‐SNP in *ABCC9* is also an expression quantitative trait locus, which affects the levels and splice variants of brain mRNA transcripts derived from *ABCC9* 17. However, in our study, LATE‐NC + HS failed to associate with *ABCC9* rs704178 genotype (evaluated both in additive and recessive mode of inheritance) or allele.

The previously reported *ABCC9* rs704178 was also not associated with TDP‐43 pathology in these cohorts. It is possible that differences in the cohort selection and case definition explain the discrepancy. It is also possible that sex could influence the findings, since men at this age are a minority of the population and power is therefore limited to test for sex differences. Findings by Nelson *et al* were not adjusted for sex 14, but given that the analyses by Katsumata *et al* were adjusted 8, this explanation is likely to be insufficient. A mechanistic link between LATE‐NC + HS and *ABCC9* variation is unclear, and this study did not replicate the previous results. Further studies on *ABCC9* and LATE‐NC + HS from different source populations are needed to confirm the lack or verify the association.

In conclusion, this study confirmed in population‐representative cohorts that *GRN* rs5848 C/T and T/T as well as *TMEM106B* rs1990622 A/A genotype are associated with loss of CA1 neurons in the aging brain, supporting a possible link through lower progranulin levels. *ABCC9* rs704178, however, was not confirmed as a genetic risk factor of LATE‐NC + HS.

## Conflict of Interest

The authors declare no conflict of interest.

## Author Contributions

*Suvi R. K. Hokkanen*: Designed this study with MK, conducted neuropathological evaluation of hippocampal sclerosis in the EClipSE cohorts, performed SNP analyses for the CC75C and CFAS cohorts, evaluated TDP‐43 pathology in half of the CFAS cohort, inter‐rater evaluated TDP‐43 pathology in CC75C and half of the CFAS cohort, conducted the statistical analyses except inter‐rater analysis, drafted and revised the manuscript. *Mia Kero*: Study design with SRKH, inter‐rater evaluated hippocampal sclerosis pathology in the Vantaa 85+ cohort, innovation and processing of PEG‐embedded blocks into FFPE‐blocks, optimization and staining of TDP‐43 staining, evaluating TDP‐43 pathology in Vantaa 85+, sequence data analysis guided by KK, preliminary statistical analyses for Vantaa 85+, drafted and revised the manuscript. *Karri Kaivola*: Gene sequencing and sequencing data analysis, guiding MK with sequencing data analysis and revised the manuscript. *Sally Hunter*: Conducted neuropathological evaluation of TDP‐43 pathology in the CC75C and half of CFAS cohort, inter‐rater evaluated TDP‐43 pathology in half of the CFAS cohort, inter‐rater evaluated hippocampal sclerosis evaluation in CFAS and CC75C, contributed to the draft and final manuscript. *Hannah A. D. Keage*: Performed the inter‐rater analysis, secured project funding, and contributed to the draft and final manuscript. *Anna Kiviharju*: Gene sequencing and sequencing data analysis in the Vantaa 85+ cohort. *Anna Raunio*: Material collection partly for sequencing, optimization and staining of TDP‐43 together with MK in the Vantaa 85+ cohort. *Pentti J. Tienar*i: Sequencing data acquisition in the Vantaa 85+ cohort, secured project funding and critical revision of the manuscript. *Anders Paetau*: Inter‐rater evaluated TDP‐43 pathology with MK in the Vantaa 85+ cohort and critical revision of the manuscript. *Fiona E. Matthews*: Secured project funding, contributed to the draft and final manuscript. *Jane Fleming:* Coordination of the CC75C study, contributed to the final manuscript. *Caroline Graff*: Provided guidance on the SNP analyses of the CFAS and CC75C cohort, contributed to the draft and final manuscript. *Tuomo M. Polvikoski*: Baseline patient material collection for Vantaa 85+ study, verified neuropathological evaluation of hippocampal sclerosis cases in the EClipSE cohort, and contributed to the draft and revised the manuscript. *Liisa Myllykangas*: Study design, critical revision of the manuscript, secured project funding, supervising Vantaa 85+ work of this EClipSE collaboration study. *Carol Brayne*: Secured project funding, contributed to the draft and final manuscript. *The EClipSE collaboration* designed the cohort studies, generated additional data, and revised the manuscript.

## Supporting information


**Figure S1.** A: Section of hippocampus showing dentate, molecular layer and CA1 from a case with severe dentate neuronal inclusions but minimal cell loss in CA1; B: CA1 from same slide showing very few pathologies immunoreactive for anti‐phosphorylated TDP‐43 antibody; C: Dentate from same slide showing a range of pathologies immunoreactive for anti‐phosphorylated TDP‐43 antibody including cytoplasmic inclusions and neurites. D: Section of hippocampus showing dentate, molecular layer and CA1 from a case with severe dentate neuronal inclusions and severe cell loss in CA1 qualifying as HScl; E: CA1 from same slide showing few pathologies immunoreactive for anti‐phosphorylated TDP‐43 antibody; F: Dentate from same slide showing a range of pathologies immunoreactive for anti‐phosphorylated TDP‐43 antibody including cytoplasmic inclusions and neurites. Scale bars: A, D = 200 μm, B, C, E, F = 50 μm.
**Table S1.** Association of GRN rs5848, TMEM106B rs1990622 and ABCC9 rs704178 with dentate TDP‐43 solid neuronal inclusions in subjects without LATE‐NC+HS.

## Data Availability

All the relevant data are within the paper and its supporting information files.
