# Supplementary figures and images for: Putative risk alleles for LATE‐NC with hippocampal sclerosis in population‐representative autopsy cohorts
Source: Brain Pathol. 2019 Aug 27;30(2):364–72. doi: 10.1111/bpa.12773 (PMC7065086; doi:10.1111/bpa.12773)

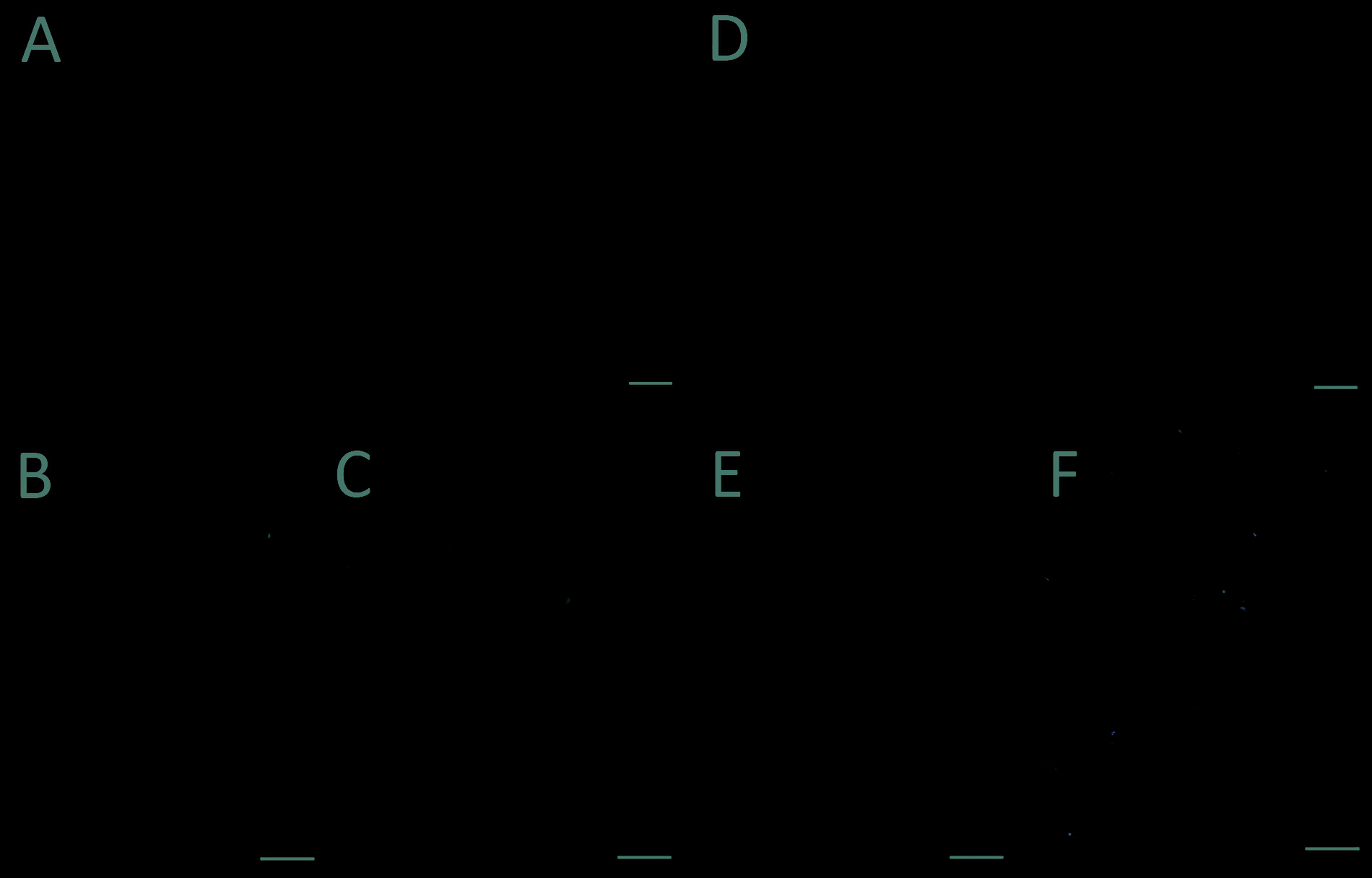

Supplement: Supplementary file 1 — Figure S1. A: Section of hippocampus showing dentate, molecular layer and CA1 from a case with severe dentate neuronal inclusions but minimal cell loss in CA1; B: CA1 from same slide showing very few pathologies immunoreactive for anti‐phosphorylated TDP‐43 antibody; C: Dentate from same slide showing a range of pathologies immunoreactive for anti‐phosphorylated TDP‐43 antibody including cytoplasmic inclusions and neurites. D: Section of hippocampus showing dentate, molecular layer and CA1 from a case with severe dentate neuronal inclusions and severe cell loss in CA1 qualifying as HScl; E: CA1 from same slide showing few pathologies immunoreactive for anti‐phosphorylated TDP‐43 antibody; F: Dentate from same slide showing a range of pathologies immunoreactive for anti‐phosphorylated TDP‐43 antibody including cytoplasmic inclusions and neurites. Scale bars: A, D = 200 μm, B, C, E, F = 50 μm. Table S1. Association of GRN rs5848, TMEM106B rs1990622 and ABCC9 rs704178 with dentate TDP‐43 solid neuronal inclusions in subjects without LATE‐NC+HS. [file BPA-30-364-s002.tif]
