# Supplementary material for: Putative risk alleles for LATE‐NC with hippocampal sclerosis in population‐representative autopsy cohorts
Source: Brain Pathol. 2019 Aug 27;30(2):364–72. doi: 10.1111/bpa.12773 (PMC7065086; doi:10.1111/bpa.12773)
Supplement: Supplementary file 2 [file BPA-30-364-s001.docx]

Supplementary Table 1. Association of *GRN* rs5848, *TMEM106B* rs1990622 and *ABCC9* rs704178 with dentate TDP-43 solid neuronal inclusions in subjects without LATE-NC+HS

|  | | No LATE-NC+HS | | | |  |
| --- | --- | --- | --- | --- | --- | --- |
|  | | No dentate TDP-43 NCI (n=535) | | Dentate TDP-43 NCI (n=122) | | Statistic |
| ***GRN* rs5848** |  |  | |  | |  |
| Genotype, n (%) | C/C | 279 | (53.9) | 48 | (39.7) | χ^2^(2)= 7.99  *p=*0.018  phi=0.112 |
|  | C/T | 190 | (36.7) | 57 | (47.1) |  |
|  | T/T | 49 | (9.5) | 16 | (13.2) |  |
| Allelic frequency, (%) | C | 72.2 | | 63.2 | | χ^2^(1)= 7.60  *p=*0.006  phi=0.077 |
|  | T | 27.8 | | 36.8 | |  |
| ***TMEM106B* rs1990622** | |  | |  | |  |
| Genotype, n (%) | A/A | 183 | (36.0) | 48 | (39.7) | χ^2^(2)= 2.06  *p=*0.358  phi=0.057 |
|  | A/G | 240 | (47.2) | 59 | (48.8) |  |
|  | G/G | 85 | (16.7) | 14 | (11.6) |  |
| Allelic frequency, (%) | A | 59.6 | | 64.0 | | χ^2^(1)= 1.59  *p=*0.208  phi=0.036 |
|  | G | 40.4 | | 36.0 | |  |
| ***ABCC9* rs704178** | |  | |  | |  |
| Genotype, n (%) | C/C | 113 | (21.1) | 21 | (17.4) | χ^2^(2)= 2.46  *p*=0.292  phi=0.061 |
|  | C/G | 277 | (51.8) | 59 | (48.8) |  |
|  | G/G | 145 | (27.1) | 41 | (33.9) |  |
| *Recessive MOI* | C/C, C/G | 390 | (72.9) | 80 | (66.1) | χ^2^(2)= 2.23  *p*=0.135  phi=0.058 |
|  | G/G | 145 | (27.1) | 41 | (33.9) |  |
| Allelic frequency, (%) | C | 47.0 | | 41.7 | | χ^2^(1)= 2.21  *p*=0.137  phi=-0.041 |
|  | G | 53.0 | | 58.3 | |  |
| LATE-NC+HS: Limbic-predominant age-related TDP-43 encephalopathy neuropathological changes with hippocampal sclerosis; TDP-43: transactive response DNA binding protein 43 kDa; n: number; MOI: mode of inheritance | | | | | | |
